# Supplementary figures and images for: CiiiDER: A tool for predicting and analysing transcription factor binding sites
Source: PLoS One. 2019 Sep 4;14(9):e0215495. doi: 10.1371/journal.pone.0215495 (PMC6726224; doi:10.1371/journal.pone.0215495)

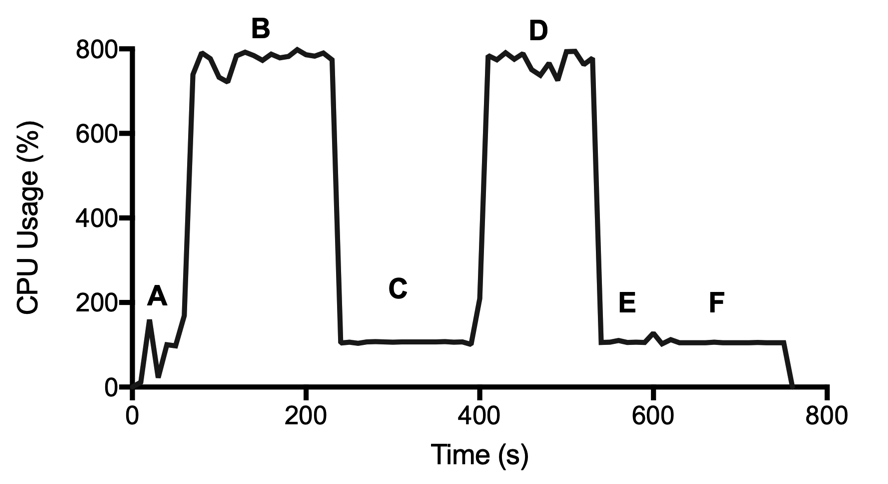

Supplement: S1 Fig — Example plot of analysis time and CPU usage of CiiiDER when performing site identification and enrichment using the Irf7 breast cancer gene set. Gene sets were loaded into the GUI and promoters were obtained (A), TFBSs were predicted across the query promoters (B) and collated (C), background sites were predicted (D), the enrichment calculation was performed (E) and the final graphical outputs were created (F). The site prediction steps take advantage of multiple computer processors. The maximum memory usage was 4.53 GB. Measurements were made on an iMac with four i7 4.0 GHz processors and 32 GB RAM. Underlying data are provided in S1 Data. (TIFF) [file pone.0215495.s001.tiff]

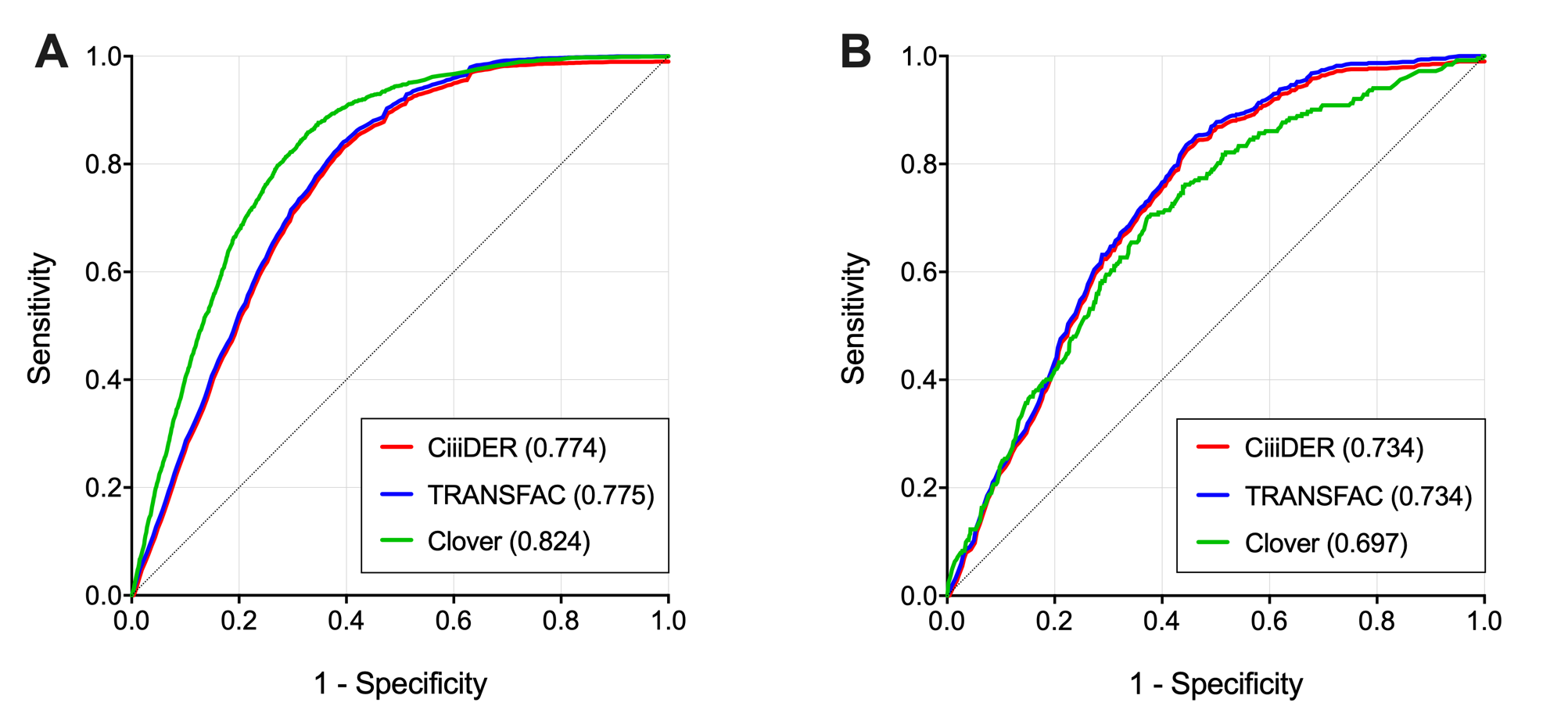

Supplement: S2 Fig — The accuracy of CiiiDER was compared with Clover and TRANSFAC software using ROC curves for (A) CTCF and (B) STAT3. The curves represent the ratio of true binding sites predicted against the number of false binding sites predicted. The locations of true binding sites have been validated previously using ChIP-seq experiments. Note that, due to almost complete overlap with the TRANSFAC curves, the CiiiDER curves for both CTCF and STAT3 were shifted down by -0.01. Underlying data are provided in S2 Data. (TIFF) [file pone.0215495.s002.tiff]
